# Thermal–Visible Face Recognition Based on CNN Features and Triple Triplet Configuration for On-the-Move Identity Verification

**DOI:** 10.3390/s22135012

**Published:** 2022-07-02

**Authors:** Marcin Kowalski, Artur Grudzień, Krzysztof Mierzejewski

**Affiliations:** 1 Institute of Optoelectronics, Military University of Technology, Gen. S. Kaliskiego, 00-908 Warsaw, Poland; artur.grudzien@wat.edu.pl; 2 Faculty of Cybernetics, Military University of Technology, Gen. S. Kaliskiego, 00-908 Warsaw, Poland; krzysztof.mierzejewski@wat.edu.pl

**Keywords:** thermal to visible face recognition, cross-spectral face recognition, biometrics, CNN

## Abstract

Face recognition operating in visible domains exists in many aspects of our lives, while the remaining parts of the spectrum including near and thermal infrared are not sufficiently explored. Thermal–visible face recognition is a promising biometric modality that combines affordable technology and high imaging qualities in the visible domain with low-light capabilities of thermal infrared. In this work, we present the results of our study in the field of thermal–visible face verification using four different algorithm architectures tested using several publicly available databases. The study covers Siamese, Triplet, and Verification Through Identification methods in various configurations. As a result, we propose a triple triplet face verification method that combines three CNNs being used in each of the triplet branches. The triple triplet method outperforms other reference methods and achieves TAR @FAR 1% values up to 90.61%.

## 1. Introduction

Face recognition has become a popular technology exploited in many aspects of our life nowadays. A significant part of the research related to face recognition explores the visible spectrum of light, while other parts of the spectrum, including near and thermal infrared, are yet to be thoroughly investigated. The main cause of this fact may lay in the cost and accessibility of the equipment required to capture thermal face images of proper quality.

Visible light imaging performs very well as long as the observed scene is properly illuminated, but this technology is not effective in low-light conditions. On the other hand, complementary thermal infrared imagery permits subjects to be observed even in pitch darkness. Combined thermal–visible face recognition may be a pivotal method to recognize subjects captured in low light conditions; however, the modality gap between thermal infrared and visible light domains needs to be filled in with the correct statistical algorithms trained using applicable databases.

This paper reports on the study and implementation of thermal–visible face recognition for identity verification of subjects on-the-move. We propose a triple triplet method to compare face images in thermal and visible domains. This study is based on the outcome of an international project under the name of “Detecting document fraud and identity on-the-fly” (D4FLY), which received funding from the European Union’s Horizon 2020 research and innovation programme. The goal of the endeavor is to provide travelers moving around border crossing facilities with effective means of identity verification. The person verification scheme comprises two stages executed in order at the enrollment kiosk and in the biometric corridor. The enrollment kiosk, equipped with thermal and visible light cameras, is used to register new subjects in the system, while the verification is performed in a few-meter-long corridor furnished with a visible light camera only.

This work’s primary contribution areas are as follows:

- Investigation of Siamese and Triplet architectures, together with VTI methods testing state-of-the-art algorithms for thermal–visible face verification;
- Examining spectral dependence of anchor images;
- Proposition of triple triplet method for using a specific set of CNNs for improved impostors and genuine subject distinction.

Section 2 describes other works in the field of thermal–visible face recognition, while Section 3 introduces the research methodology. Datasets are described in Section 4, followed by the overview of investigated methods in Section 5. We present results and summary in Section 6 and Section 7, respectively.

## 2. Related Works

In this section, we present works related to visible and cross-spectral face recognition between thermal infrared and visible face images, specifically for subject identity verification.

Face recognition has gained superior popularity mainly in the visible spectrum due to easily accessible sensors integrated with popular electronic devices, including but not limited to smartphones and laptops. Current face recognition systems can outperform human perception capabilities in the visible spectrum of light, and are deemed the foremost tools available.

Taigman et al. [1] proposed the DeepFace method, which effectively generalized face representation to other datasets. They proposed a new deep neural network architecture and learning method, which achieved an accuracy of 97.35% when evaluated on the LFW dataset.

Wen et. al. [2] proposed center loss as a new loss function to enhance the discriminative power of the deeply learned features in neural networks. It allows for minimizing intraclass distances of the deep features. They achieved an accuracy of 99.28% while evaluated on an LFW dataset.

Parkhi et al. [3] introduced a very large-scale dataset VGG Face and investigated various CNN architectures. They were inspired by VGG architectures. They showed that the training process and a dataset are the key factors for the face recognition method. They also showed that it is possible to achieve 98.95% accuracy with the appropriate training process.

Liu et al. [4] proposed an angular Softmax loss for CNNs to learn discriminative face features with an angular margin. Their work presents a deep hypersphere embedding approach. Their proposed A-Softmax loss is very effective for learning face representation and achieved an accuracy of 99.42%.

On the contrary, thermal–visible face recognition is not as popular and widely studied as methods grounded in the visible domain, and results reported in this modality combination are lower in performance. One of the initial works in this field is focused on thermal–visible face identification [5]. Hu et al. proposed a method based on a specific pre-processing stage, Histogram of Oriented Gradients and Partial Least Squares based model. The proposed pre-processing stage consists of four components applied in the following order: median filtering of dead pixels, geometric normalization, difference-of-Gaussian (DOG) filtering, and contrast enhancement. Evaluation of the proposed method is conducted using the University of Notre Dame (UND) Collection X1, a dataset collected by the Wright State Research Institute (WSRI), and a dataset acquired by the U.S. Army Night Vision and Electronic Sensors Directorate (NVESD). They performed experiments at distances from 1 to 4 m, also analyzing the impact of the exercises on the results of thermal-to-visible identification. The results they obtained for the Rank-1 Identification metric: 0.7 (equivalent to 70%) for the distance of 1 m before exercise, and 0.64 after exercise (equivalent to 64%).

Chen et al. [6] presented face recognition between visible and thermal images based on a cascaded subspace learning scheme. This scheme is composed of whitening transformation, factor analysis, and common discriminant analysis. They used a factor analysis model to extract the identity factor as a subject across different spectra. In order to reduce some of the cross-spectral appearance differences, they tested filtering algorithms, such as Center-Surround Divisive Normalization (CSDN) and Self Quotient Image (SQI). After the application of image filters, the Pyramid Scale Invariant Feature Transform (PSIFT) and Histograms of Principal Oriented Gradients (HPOG) descriptors are utilized to extract features from thermal and visible face images. The application of whitening transformation is to ensure that the distribution of samples conforms to an isotropic Gaussian as required by the Hidden Factor Analysis. The decision function is based on Partial Least Squares (PLS) and Canonical Correlation Analysis (CCA). They used the PCSO dataset as a training dataset and performed face matching between visible and thermal face images using the CARL dataset. The final results, combining the two feature extraction methods by means of fusion, gave a score of 75.61% for the Rank-1 Identification rate, 27.71% for the 0.1% FAR, and 51.24% for the 1% FAR in the verification approach.

Sarfraz et al. [7] presented a study to find a bridge between the two modalities by trying to model directly the highly non-linear mapping. The authors developed a model based on a feedforward deep neural network in order to map the perceptual differences between the two modalities while preserving the identity information. The study was based on the University of Notre Dame UND-X1 dataset. They achieved an accuracy of 83.73% using all visible images in the gallery for the Rank-1 Identification rate, and 55.36% when they were used in the gallery one visible face image per subject.

In [8], Kantarci et al. showed mapping between thermal and visible domains using a deep autoencoder model. They used the UND-X1, CARL, and EUROCOM cross-spectral datasets to evaluate the performance of the proposed system. Their study showed that deep convolutional autoencoders can learn non-linear mapping between thermal and visible images for the cross-domain face recognition task. As a decoder, they used two different upsampling methods. The first one is bilinear upsampling, which is a standard interpolation technique. This approach reduces the number of trainable parameters and decreases training time on a GPU dice, but as information for the decoding part is lost, the performance is degraded. The second method is a convolution with a 2 × 2 filter size as proposed in the U-Net. The implemented loss function for this network is mean square loss, so it makes its output as similar as possible to the ground truth thermal images. For the CARL dataset, they achieved the best scores of 48% for the Rank-1 Identification rate when one visible image per subject is in the gallery, and 85% when all images per subject are available. The study was repeated using the UND-X1 dataset and achieved 87.2% accuracy for all images in the gallery and 58.75% for the Rank-1 identification rate for one visible image in the gallery. For the EUROCOM dataset, the results are the following: 88.33% and 57.91% for all images per subject and one image per subject in the gallery, respectively.

Fondje et al. [9] proposed a domain adaptation framework, which consists of feature extraction using a truncated deep neural network for visible and thermal face images, Residual Spectral Transform (RST) between thermal and visible features, cross-domain identification loss, and domain invariance loss. Features are extracted using VGG16 and ResNet-50 architectures. The RST is a residual block that allows as much discriminability from the truncated networks as possible to be preserved while transforming features between thermal and visible domains. Before conducting all stages from the proposed framework, they applied the Difference of Gaussians filter to visible and thermal face images. For testing, they used three separate datasets/protocols compiled by the CCDC Army Research Laboratory. For frontal face images, they achieved a Rank-1 Identification rate of 96% and 84% for ResNet-50 and VGG16, respectively.

Numerous works in this field have been performed using generative adversarial networks (GAN) to transform an image of one modality into an image in the second domain.

Thermal–visible face recognition has also been addressed by employing GAN networks. In [10], Mallat et al. proposed image synthesis for cross-spectrum face recognition, consisting of generating visible-like images from thermal captures that will be matched against a gallery of visible faces. Cascaded refinement networks coupled with contextual loss allow high-quality-colored visible images to be synthesized from thermal acquisitions. They used their own EUROCOM dataset to test the proposed method. Accuracy of face recognition achieved 20% for neutral expressions of face images using OpenFace. For the LightCNN system, they achieved 82% accuracy for neutral face images. The major drawback of the GAN-based methods is a long processing time, which does not allow them to be used for on-the-move recognition.

Wang et al. [11] developed a model consisting of a generative network based on the CycleGAN and detector network. The GAN network learns the bidirectional translation between thermal and visible images based on an unsupervised manner using unpaired training images. The detection network extracts 68-landmarks from visible faces, constructing the shape loss function and helping the optimization of the generative network. They used for research their own dataset consisting of 792 aligned thermal and visible image pairs of 33 subjects. Images were taken by camera FLIR AX5. From generated probe and gallery images were obtained features using the Facenet toolbox. Next, they used Euclidean distance between features of the probe and features of the gallery. The shortest distance was taken to predict matching and to determine whether it is correct or not. They achieved 91.6% for the Rank-1 rate using the Facenet method for their own generated thermal and visible images.

Kezebou et al. [12] proposed a framework to automatically synthesize visible face images captured in the thermal domain, called TR-GAN (thermal to RGB Generative Adversarial Network). TR-GAN is based on U-Net architecture with cascade residual blocks for a generator. the generator synthesizes images with consistent global and local structural information. They used a pretrained VGG-Face recognition model and ResNet-50 to perform the face comparison after the thermal to visible image translation. The study was conducted using a TUFTS dataset. For the ResNet-50 model, they achieved 80.7% accuracy of identification, and the VGG16 accuracy of identification was 88.65%.

Immidisetti et al. [13] proposed an Axial GAN framework to synthesize high-resolution visible images from low-resolution thermal images. Their framework is characterized by an axial-attention layer. An axial layer effectively captures long-range dependencies with high efficiency. The study was performed using an ARL-VTF dataset. They used cosine similarity between features extracted from a VGG-Face model and achieved an AUC of 91.23%.

Anghelone et al. [14] proposed a Latent-Guided Generative Adversarial Network (LG-GAN) to decompose images into an identity latent code and a style latent code. It allows spectral-invariant and spectral-dependent properties to be obtained. LG-GAN can preserve the identity during the spectral transformation and achieve face recognition results with respect to a visual quality of 96.96% for the AUC rate. For testing purposes, they used cosine distance between features extracted from ResNet-50.

The work of Cao et al. [15] presents a conversion of a visible face image into a thermal face image (V2T) and a thermal face image into another one with a different temperature of the face (T2T). They developed a framework based on a U-Net generator and a six-layer PatchGAN discriminator. To conduct the V2T task they used the Speaking Face Database, and for the second task (T2T), the Carl database was used. The model is trained using a combination of cGAN loss, perceptual loss, and temperature. This work combines two fields of cross-spectral recognition, including thermal to thermal recognition and thermal to visible for generated and real images from each spectrum. The face recognition task is based on three pretrained models: InceptionV3, Xception, and MobileNet. They used pretrained weights optimized for the ImageNet database for each neural network. They removed the last fully-connected layer and classification layer from each model and added an average pooling layer, two fully-connected layers with 512 units, and a classification layer to each model. Finally, the method achieved about 78% for Rank-1 tested on the Speaking Face database, and about 96% for Rank-1 rate tested on the Carl database.

Poster et al. [16] have developed their own thermal to visible face images database. This database consists of 395 subjects. The total number of images is equal to 549,712. The distance subject between the camera is 2.1 m. Visible face images were acquired using an RGB Basler Scout CCD camera and thermal face images were acquired using an FLIR Grasshooper3 CMOS camera. For the purpose of face recognition, they used five different methods. Four methods are based on the GAN framework including Pix2Pix [17], GANVFS [18], SAGAN [19], and “Raw”, which is a naïve baseline method. Thermal images (probes) and visible images (gallery) were provided directly to the VGG-Face model with a cosine similarity measure. The results are 2.77%, 6.95%, 6.69%, 84.88%, 91.55%, and 96% for RAW, Pix2Pix, GANVFS, SAGAN, and Fondje’s method, respectively.

## 3. Methodology

For this study, we developed a methodology that consists of data collection rules, algorithms selection, and experiment design. This multi-step methodology aims to develop several algorithms starting from dataset preparation and annotation, pre-processing, and face detection up to the face verification process, corresponding to the development of the feature extraction methods and decision functions.

Since the extraction of features is proposed to be reused in other face verification architectures, several neural networks have been trained for identification purposes. These networks are the basis for feature extraction in various architectures considered during this study.

The following is a brief overview of the algorithm development phases:

(1) Database preparation and annotation. In this step, the datasets are combined and divided into training and testing splits. Another division used in this paper corresponds to the number of subjects and the presence of glasses. Since glasses are not transparent in the thermal infrared domain, they may impact the performance of thermal–visible face recognition. We decided to split the database according to the presence of glasses to assess this impact.
(2) Development of face detectors. Wide study has been performed in this context to evaluate the possibility of using a single face detector for both spectrums. As a result, we developed two separate face detection algorithms for thermal and visible images, respectively.
(3) Training of CNNs for identification and feature extraction. The selected CNNs have been trained for classification purposes to learn the feature representation of thermal and visible images. The training procedure comprises the following steps:
  - Pre-training of CNNs with the ImageNet database;
  - Training of all CNNs with a joint database of visible and thermal images;
  - Training of all CNNs with separate databases of visible and thermal images.

The trained CNNs will be further re-used for feature extraction in all the studied architectures.

(4) Development and testing of various methods including:
  - Siamese-based methods;
  - Triplet-based methods;
  - Verification through identification approach.
(5) Analysis and selection of the best algorithm. This step corresponds to the calculation of performance metrics and the speed of processing.

All the experiments have involved various settings of databases. Another goal was to evaluate how the data quality may impact the algorithm performance, especially for thermal infrared images.

In order to validate the developed method in a controlled environment, it has been agreed that the thermal–visible face recognition system will be distributed across two components of the D4FLY system: an enrolment kiosk and a biometric corridor.

(a) A thermal infrared sensor will be embedded into the enrolment kiosk;
(b) The acquisition of face images on the move will be performed in the biometric corridor using a visible light camera.

The proposed architecture was implemented in the D4FLY prototype and tested during multiple field test events.

## 4. Datasets

For the training, evaluation, and testing, several datasets were used during this study. The main requirement for all databases was to include respective images of faces acquired in the visible domain and thermal infrared. The datasets used for development came from our own repositories and from external sources. In this section, we briefly describe all the datasets that have been utilized during the study.

### 4.1. D4FLY Thermal and 2D Face

**Figure 1:**
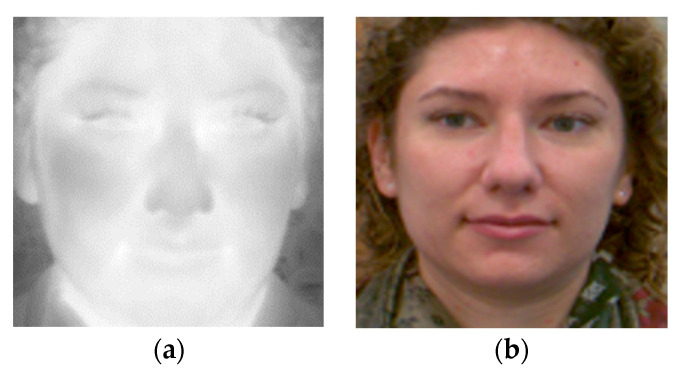
Sample face images from the D4FLY dataset: (**a**) thermal infrared; (**b**) visible spectrum.

The dataset consists of images of 31 subjects. Face images were acquired for each person at a distance between 1.5 and 4 m from the camera. After the subject had come towards the camera and stopped, the images were taken of the face in different head positions, but mainly in the frontal position (Figure 1). Images were captured using Basler acA2040-90uc (resolution of 2040 × 2046 pixels) and FLIR A65 cameras (resolution of 640 × 512 pixels).

### 4.2. IOE_WAT Dataset

**Figure 2:**
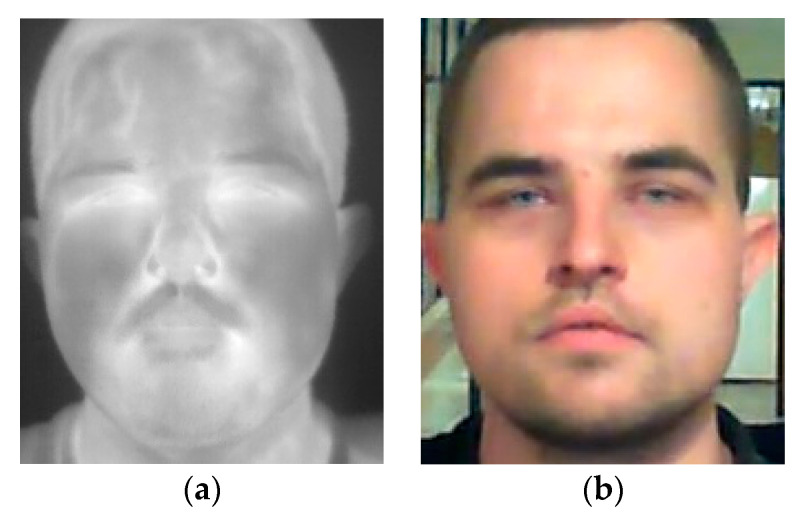
Sample face images from IOE_WAT dataset: (**a**) thermal infrared; (**b**) visible spectrum.

This dataset was collected at the premises of the Military University of Technology and contains visible and thermal infrared images of 40 subjects [20]. Visible images were acquired using a Microsoft webcam camera and Microsoft Kinect v2 with resolutions of 1280 × 720 pixels and 1920 × 1080 pixels, respectively (Figure 2). Thermal infrared images were acquired using FLIR A65 (for 16 subjects) and FLIR P640 (for 24 subjects), both with a resolution of 640 × 512 pixels and an NETD below 50 mK. During the acquisition process, the subject was sitting in front of the camera at a distance of 1.5 m and more than 100 images were collected for each of the subjects. This dataset contains images of 12 subjects wearing glasses.

### 4.3. Speaking Faces Dataset

**Figure 3:**
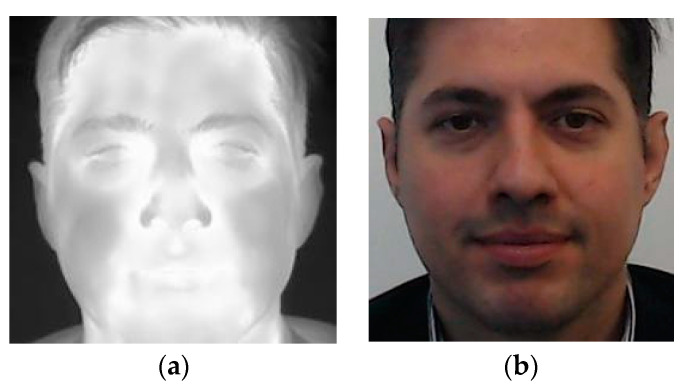
Sample face images from the Speaking Faces dataset: (**a**) thermal infrared; (**b**) visible spectrum.

Dataset [21] is a publicly available large-scale multimodal dataset that contains thermal, visual, and audio data of 142 different subjects. Visual, thermal, and audio data were collected from the same nine camera positions during two sessions. During the first session, the subjects were silent, as opposed to the second session when they were asked to read a series of sentences. Subjects were directed in front of the camera at approximately one meter (Figure 3). Thermal images were acquired using an FLIR T540 camera with a resolution of 464 × 348 pixels while the visible images were captured using a Logitech C920 Pro HD web-camera with a resolution of 1920 × 1080 pixels.

### 4.4. Sejong Face Dataset

**Figure 4:**
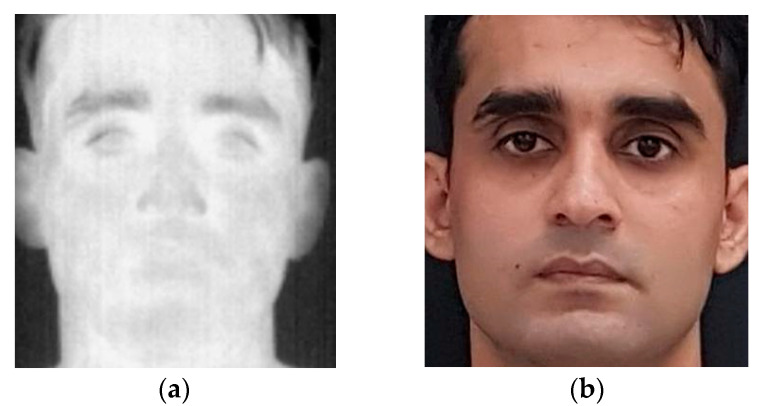
Sample face images from Sejong Face dataset: (**a**) thermal infrared; (**b**) visible spectrum.

The Sejong dataset [22] consists of two subsets (subsets A and B). Subset A contains face images of 30 subjects (14 males and 16 females) while subset B contains face images of 70 subjects (44 males and 26 females). The dataset includes images captured in special conditions including subjects with glasses, masks, beards, fake beards, or scarf. The visible images were captured using a smartphone device with a resolution of 4032 × 3024 pixels (Figure 4). The thermal face images were captured using a Therm-App camera. The resolution of images captured using a thermal camera is 768 × 756.

### 4.5. FaceScrub Dataset

**Figure 5:**
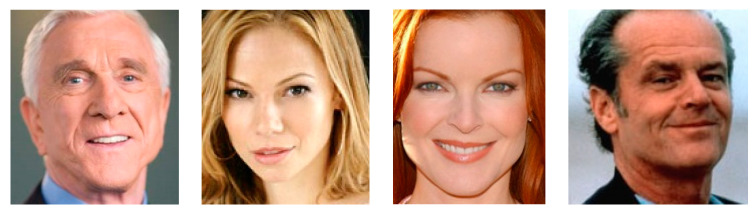
Sample visible face images from the FaceScrub dataset.

This dataset [23] is a collection of unconstrained visible images of 530 subjects (Figure 5). Each subject is captured at around 200 images, which corresponds to a total number of 106,863 images. This dataset is equally divided into male and female subjects. The resolution of images differs across the whole dataset.

### 4.6. Training and Testing Dataset

The datasets described in Section 4.1, Section 4.2, Section 4.3, Section 4.4 and Section 4.5 have been used either for training or testing purposes. During the training process, we used 80% of the images from the randomly selected subjects from the D4FLY and IOE_WAT databases, which will be further referred to as the joint dataset. The joint training dataset contains images of 71 subjects. Each subject is represented by 24 images, equally distributed between visible and thermal infrared domains. Eight images of each subset present the face in frontal position, and four images present the face turned into right, left, up, or down. Images of subjects wearing glasses were not included in the training dataset.

The testing dataset corresponds to the remaining 20% subjects of D4FLY and IOE_WAT supplemented with the first package of the Speaking Faces dataset and selected images of the Sejong Face dataset. Due to the large rotation of the face images in the Sejong Face dataset, only five frontal face images for each subject were used.

The testing dataset was divided into two subsets composed of images presenting subjects with and without glasses. Finally, the number of subjects wearing glasses was 46, and not wearing glasses was 96. 

As part of the study concerns the use of the VTI method [24], a set of image doublets was prepared. We prepared a set of doublet images from previously grayscale converted RGB images and thermal infrared images.

During this study, data augmentation techniques were applied for each of the datasets. Each dataset was composed of an equal number of images from thermal infrared and visible light domains.

### 4.7. Assessment of Data Bias

Since the study covers the face recognition task, it is important to balance the datasets to avoid or minimize the impact of various types of bias. The datasets used during this study were characterized in data bias terms. The statistical information on subject gender distribution is provided in Table 1. As it can be observed, male subjects stand for the majority of all samples.

As the datasets are not annotated with geographical information, we are unable to provide statistical information on the race distribution of the subjects.

## 5. Proposed Method

The study pursues a face recognition method that allows on-the-move object recognition with respect to the execution time. The GAN-based methods reported in the literature are computationally expensive and slow; therefore, we decided to apply the CNN-based approach. As a starting point, we prepared the face detection algorithm. Due to the modality gap between visible and thermal infrared images, we developed two separate face detection algorithms for each respective modality. As a result of our previous investigations, we used the Faster R-CNN with ResNet-101 to train two separate models. Study on the thermal face detection process is described in [25].

### 5.1. Triple Triplet Method-Overview

We propose a modified triplet-based algorithm called triple triplet for thermal–visible face recognition. The standard triplet architecture requires three images to be processed simultaneously using analogous processing paths to compute the final scoring. In general, the three images are called positive, negative, and an anchor. The idea behind using an anchor image is to increase the separation of similar and dissimilar face images.

Since the considered study uses two different modalities, the anchor image may be either visible or thermal infrared. The most popular trend in the literature indicates that the anchor should be of the same class as the positive image. Our in-depth study showed that the anchor image should be in the visible domain.

Each of the processing paths in the triplet architecture uses a similar CNN model, but in our case, each CNN is trained either on thermal or visible images, depending on the spectral range of the processed images. After the feature extraction stage, feature vectors are used to calculate the distance *D* between them. Several existing distance functions were considered to calculate similarities between vector correlation distance, Spearman distance, Euclidean distance, and city distance (L1 distance). Moreover, we considered a triplet loss function, which can be calculated by the following formula:

(1) $$D_{triplet}=D\left(f\left(P\right),f\left(A\right)\right)-D\left(f\left(N\right),f\left(A\right)\right),\;$$

where *D*(*f*(*P*),*f*(*A*)) is a distance function calculated between feature vectors of a positive image *f*(*P*) and an anchor image *f*(*A*), and *D*(*f*(*N*),*f*(*A*)) is the distance between the feature vectors of a negative image *f*(*N*) and an anchor image *f*(*A*).

**Figure 6:**
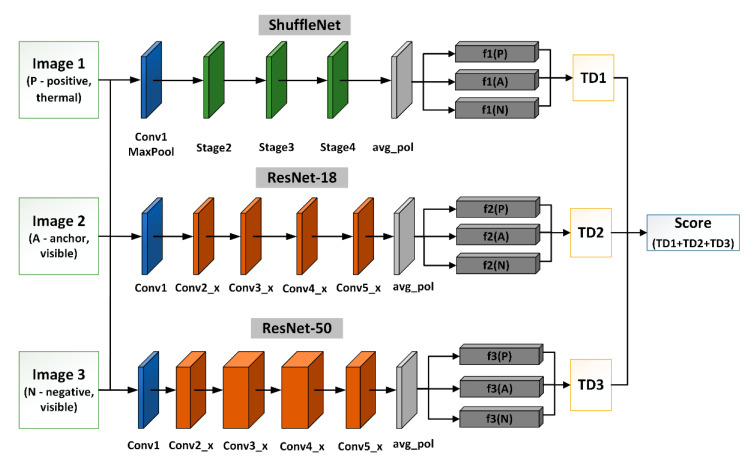
Block diagram of triple triplet architecture.

In this paper, we propose a triple triplet method by adding two additional convolutional neural networks into each branch of the triplet architecture. As a result of this modification, each branch uses three different CNNs to compute feature vectors simultaneously. These networks, namely, ShuffleNet, ResNet-18, and ResNet-50, aim to extract features from three face images at the same time, as presented in Figure 6. We assumed that due to the different numbers of network layers and different types of networks, the extracted feature vectors should differ for each network. The main difference should result from the differences in the lower-order features, i.e., those that do not relate to the main edges of the face (higher-order features) and may be associated with lines on the face and side edges. Since higher-order features are extracted at the initial layers of the neural network, the differences in the characteristics of the features will be related to the end layers of the neural networks.

The proposed method generates nine feature vectors as a result of the feature extraction process. We have noticed that to achieve the highest performance, features should be extracted from the layer preceding the fully connected layer, which, in the case of the CNNs that we were employing, corresponds to the average pooling layer. Figure 6 shows the architecture of a triple triplet with three neural networks.

As a next step, all feature vectors are used to calculate triplet distances (*TD*1, *TD*2, the *TD*3) using the same formula as in (1). The triplet distances are calculated for each of the CNNs by the following formulas:

(2) TD1=D(f1(P),f1(A))−D(f1(N),f1(A)), 

(3) TD2=D(f2(P),f2(A))−D(f2(N),f2(A)), 

(4) TD3=D(f3(P),f3(A))−D(f3(N),f3(A)), 

where the indices of feature vectors correspond to the number of the CNN used for the feature extraction. Finally, the scoring is calculated based on the triplet distances according to the formula:

(5) Score=TD1+TD2+TD3

The final score is a value that can be used to determine the decision threshold. By employing three CNNs instead of a single CNN, we were able to achieve larger separation between positive subjects and impostors. This means that the similarity between images from the same subject increases while dissimilarity between images from different subjects decreases. Triplet configurations with two neural networks were also tested, providing lower performance than the proposed solution.

### 5.2. Training Process

The proposed and reference methods were trained with the joint database described in Section 4. For the reference, Siamese-based, triplet-based, and the VTI methods were used. All the methods were implemented in a MATLAB 2021b environment with an NVIDIA RTX 2080 GPU-powered processing unit. The main toolboxes used during experiments include the Deep Learning Toolbox, Computer Vision Toolbox, and Image Processing Toolbox. The training process relies on transfer learning with state-of-the-art CNNs. We used pretrained weights from models trained on the ImageNet database for each neural network considered in this study, including AlexNet, DenseNet-201, GoogLeNet, InceptionV3, MobileNetv2, ResNet-18, ResNet-50, ResNet-101, ShuffleNet, SqueezeNet, VGG16, and VGG19.

In the first step, all CNNs were trained using the FaceScrub database with a split ratio of 90% to 10% for training and validation subsets, respectively. Before the training process, the Softmax and the last fully connected layers were removed and replaced by new layers of the same type with the number of neurons correlated to the number of subjects in the FaceScrub dataset. The learning parameters set during the training process can be found in Table 2 below. The training process was continued until the classification accuracy calculated for the validation subset did not improve for 20 network validations in a row. All values and parameters were determined empirically.

Each of the trained models was used to extract features in the Siamese-based, triplet, and triple triplet methods. To conduct experiments based on the VTI method, we used the same neural network models pretrained on the ImageNet database. Since the VTI method outputs two classes (genuine and impostor), the number of neurons in the final Softmax layer equals two.

In the final step, all the CNNs were trained using 80% of the subjects from the D4FLY and IOE_WAT datasets. All the datasets were gathered in the data stores.

### 5.3. Testing Experiments

Testing experiments were divided into two parts, corresponding to subjects wearing and not wearing glasses. We used different datasets during each of the two experiments because of the availability of data presenting subjects wearing glasses. A composition of IOE_WAT, D4FLY, and Speaking Faces databases was used for testing the algorithms for subjects not wearing glasses, while the IOE_WAT, Speaking Faces, and Sejong Face databases were used for subjects with glasses.

## 6. Results

In this section, we present results divided into four parts corresponding to models and architectures developed and tested during the study. All the results are calculated using well-known biometric rates including the True Acceptance Rate (TAR) and False Acceptance Rate (FAR) of 1% and 0.1%, respectively. The overall dependence of TAR and FAR values is described by the ROC characteristics. During the tests, we calculated similarities between the feature vectors using various distance functions including cosine, Spearman, correlation, and Euclidean to compute the final face verification score.

### 6.1. Siamese Networks

The first part of the study concerns using Siamese architecture. Table 3 presents the results for three models that achieved the best TAR values. The best TAR is achieved by ResNet-50 with a Spearman distance. We observed a significant increase in FAR and a reduction in TAR for images presenting face images of subjects wearing glasses.

All the methods achieve better results for subjects wearing glasses, with the highest scores obtained for the joint dataset. The values of performance metrics achieved by the methods based on the Siamese architecture are far below expectations.

### 6.2. Triplet Networks

In the second part of the study, we investigated the triplet-based methods in various configurations. We conducted two experiments corresponding to two cases. In the first experiment, we used thermal infrared images as anchors. The results of the experiment are presented in Table 4.

During the second experiment, we used visible images as anchors. The results of the second experiment are presented in Table 5. The best performing CNNs include the ResNet-18 visible anchor image and Inceptionv3 for thermal anchor images.

The overall performance of the methods based on the triplet architecture is higher than the Siamese methods. The presented results indicate that the best results were obtained when the visible images were used as an anchor. Moreover, the TARs are mostly similar along different datasets with the best results achieved for the Speaking Faces database.

### 6.3. Verification through Identification

Since the Verification Through Identification method performed very well in the thermal-to-thermal face verification task, the method was considered as a part of this study. The results obtained with the three best configurations of the VTI method are presented in Table 6. The best TAR values were achieved with the Inceptionv3-based algorithm, and they are the lowest achieved values of all the studied methods.

### 6.4. Triple Triplet

As an outcome of the results that we achieved with the reference methods, we proposed a modification to the best-performing triplet-based algorithm, as presented in Section 5.1. Based on the experiments with triplet-based algorithms, our proposed algorithm always uses a visible anchor image. During the study, we also considered the architecture with two CNNs instead of three, with various CNNs for feature extraction and various learning parameters. Cumulative results of the method are presented in Table 7.

**Figure 7:**
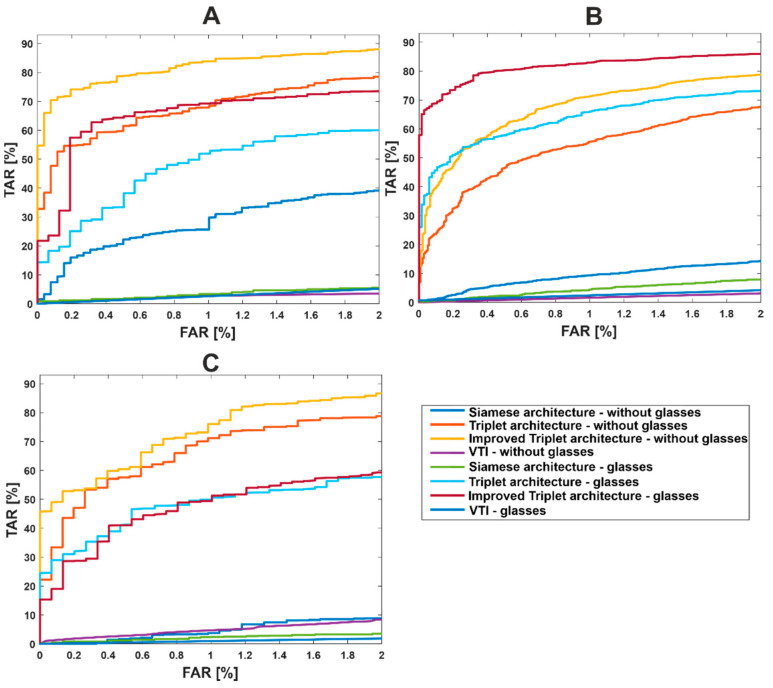
ROC curves for each of investigated approaches using the joint database (**A**), Speaking Faces (**B**), and Sejong Face (**C**).

Figure 7 presents ROC curves of all investigated algorithms divided by the dataset used for testing purposes. The presented graphs indicate the data bias, which is a result of imbalanced and relatively small datasets.

For the two best-performing methods, we prepared an additional analysis showing the impact of a head position on identity verification. The results are presented in Table 8. During this analysis, the Sejong Face database was not used because it does not contain images of the subjects with rotated head. We can observe an increase in the TAR value for rotated face images between the results for the triple triplet method compared to the standard triplet architecture. For example, TAR for the Speaking Faces dataset with subjects not wearing glasses increased from 77.81% to 88.89%.

### 6.5. Data Biases-Gender

Referring to Section 4.7, we composed a sub-study related to the data biases. In Table 9 and Table 10, we present results achieved by the triplet-based and the proposed triplet-based algorithm, respectively. The two tables present the TAR values of the best algorithm configurations. All the results presented in the tables are calculated for subjects not wearing glasses.

The results presented in both tables show disproportion of the algorithms’ performance between male and female subjects. Moreover, the TAR value differences are also visible between the different testing databases. Both the gender and dataset bias are the result of relatively small and unbalanced datasets, which should be supplied with more subjects of both sex and uniform age distribution.

### 6.6. Processing Time

The study aims to look for the thermal–visible face recognition methods that are able to process the images while the subject is walking through the biometric corridor. This condition is strictly connected to the processing speed. The average processing time of the proposed method is presented in Table 11. Time is calculated using the sum of the following components: time of loading images, time of extracting features, and time of decision. We present time for two methods. Verification time using the triple triplet method is about two times greater than during using the triplet method. Our method uses three models; therefore, feature extraction is performed three times longer, which increases the total time of the verification process.

### 6.7. Ablation Study

In this section, we present the results of the method being trained without an essential part of the data. For this part of the study, we selected the two best-performing methods and trained them with single domain images. First, the methods were trained with a dataset containing visible face images. In the second step, the same methods were trained with thermal infrared images only. The hyperparameters of the training process are the same, as presented in Table 2.

In both cases, the methods were evaluated with a testing dataset composed of thermal infrared and visible images. The results of the ablation study are presented in Table 12.

## 7. Conclusions and Future Works

Cross spectral face recognition using thermal and visible face images is not a well-explored field of research, mainly due to high-priced equipment and the relatively limited number of free image databases ready for use. Numerous manuscripts have reported the results found in the field of GAN methods, which can generate visible images based on thermal infrared imagery. However, these methods are not suitable for on-the-move verification due to their long processing time.

In this work, we present the results of our study in the field of thermal–visible face verification using four different algorithm architectures with a proposed triple triplet method, which combines three CNNS being used in each of the triplet branches.

In our study, we investigated several different algorithms in various configurations including the Siamese, Triplet, and VTI methods. For the training and testing purposes, we used several publicly available datasets composed of corresponding thermal and visible domain images.

Since all the methods that we investigated used the same CNN models for feature extractions trained in the same conditions and configurations, the presented results are easily comparable. Our proposed triple triplet method achieves TAR @FAR 1% values up to 90.61%, depending on the testing dataset. Compared to the traditional triplet-based method, the triple triplet method increases the TAR value from about 65–67% to 73–77% when tested on the joint dataset. Compared to related works in the field, the proposed triple triplet outperforms the corresponding methods. Most of the works listed in the related works section are focused on the identification task, and thus are not directly comparable with our undertaking. The work of Chen et al. [6], focused on the verification task, presents a TAR of 51.24% for 1% of FAR, while in [15], the GAN-based method tested on the Speaking Faces database achieved a Rank-1 of 78%.

All the algorithms were fourfold tested for performance, partitioned by an attribute related to glasses put on or removed, and another for frontal or rotated head positions. As anticipated, all the methods achieve better results for subjects not wearing glasses, since glasses are not transparent in the thermal infrared domain and significantly reduce the number of facial features when being worn. We also noticed a large variation in the results when testing with different datasets. Since the joint dataset was used during the training process, this fact may indicate a hardware bias, or a dataset bias in general, which is a result of a relatively small training dataset.

The presented study shows that there is still a lack of publicly available large datasets of joint thermal infrared and visible face images. The quite high FAR values can be overcome when using large datasets during the training process. In addition, the study showed that glasses lower the method’s performance; thus, subjects should be asked to remove them before being checked.

The presented study is considered as another step toward a fully functional thermal–visible face recognition method. In future works, we want to expand our dataset and propose new algorithms and methods.

## Figures and Tables

**Table 1:** Gender split of datasets.

	Gender	
Name of Dataset	Female	Male
Joint dataset (training)	36%	64%
Joint dataset (testing)	56%	44%
Speaking Faces	48%	52%
Sejong Face	33%	67%

**Table 2:** Learning parameters set during the training process.

Name of Parameter	Value/Method
Learning rate	0.0003
Batch size	64
Optimization method	stochastic gradient descent with momentum (SGDM)

**Table 3:** Performance of Siamese algorithms with the joint ^1^ dataset, Speaking Faces dataset, and Sejong Face dataset.

Joint Dataset ^1^					
CNN Model	Distance Function	Without Glasses		With Glasses ^2^	
		TAR@ FAR 0.1% ^3^	TAR@ FAR 1% ^3^	TAR@ FAR 0.1% ^3^	TAR@ FAR 1% ^3^
MobileNetv2	Cosine	21.10 (FAR = 0.66)	40.82 (FAR = 2.85)	21.65 (FAR = 6.63)	56.94 (FAR = 20.33)
ResNet-50	Spearman	18.48	43.40 (FAR = 1.66)	4.04 (FAR = 1.20)	21.53 (FAR = 6.44)
VGG19	Spearman	16.47 (FAR = 0.23)	42.67 (FAR = 3.09)	9.85 (FAR = 1.26)	33.08 (FAR = 8.40)
**Speaking Faces Dataset**					
**CNN Model**	**Distance Function**	**Without Glasses**		**With Glasses**	
		**TAR@** **FAR 0.1% ^3^**	**TAR@** **FAR 1% ^3^**	**TAR@** **FAR 0.1% ^3^**	**TAR@** **FAR 1% ^3^**
MobileNetv2	Cosine	16.99 (FAR = 1.19)	36.88 (FAR = 4.36)	4.92 (FAR = 1.65)	22.78 (FAR = 10.10)
ResNet-101	Correlation	23.06 (FAR = 3.96)	50.95 (FAR = 12.43)	2.85 (FAR = 0.62)	18.19 (FAR = 6.37)
VGG19	Spearman	13.43 (FAR = 1.61)	37.46 (FAR = 7.60)	5.09 (FAR = 0.91)	28.79 (FAR = 8.32)
**Sejong Face Dataset**					
**CNN Model**	**Distance Function**	**Without Glasses**		**With Glasses**	
		**TAR@** **FAR 0.1% ^3^**	**TAR@** **FAR 1% ^3^**	**TAR@** **FAR 0.1% ^3^**	**TAR@** **FAR 1% ^3^**
ResNet-18	Correlation	2.49 (FAR = 0.66)	12.82 (FAR = 3.21)	0.87	10.06 (FAR = 6.30)
VGG16	Correlation	6.10	12.00 (FAR = 1.77)	0.13	4.63
VGG19	Spearman	7.15 (FAR = 0.59)	6.82	4.83	29.51 (FAR = 6.51)

^1^ This dataset contains the IOE_WAT and D4FLY datasets. ^2^ This is the only IOE_WAT dataset that contains subjects wearing glasses. ^3^ The values located in parentheses indicate the FAR calculated for the test dataset only if it differs significantly from the FAR calculated for the training dataset.

**Table 4:** Performance of Triplet algorithms for the joint ^1^, Speaking Faces, and Sejong Face datasets for a visible anchor image.

Joint Dataset ^1^					
CNN Model	Distance Function	Without Glasses		With Glasses ^2^	
		TAR@ FAR 0.1% ^3^	TAR@ FAR 1% ^3^	TAR@ FAR 0.1% ^3^	TAR@ FAR 1% ^3^
ResNet-18	Cosine	46.99	65.74	29.23	53.47
ResNet-50	Cosine	43.71	67.90	27.59	56.12 (FAR = 1.83)
ShuffleNet	Cosine	40.70	68.21	18.31	51.45
**Speaking Faces Dataset**					
**CNN Model**	**Distance Function**	**Without Glasses**		**With Glasses**	
		**TAR@** **FAR 0.1% ^3^**	**TAR@** **FAR 1% ^3^**	**TAR@** **FAR 0.1% ^3^**	**TAR@** **FAR 1% ^3^**
MobileNetv2	Cosine	45.31 (FAR = 0.50)	67.09 (FAR = 2.01)	41.38	67.83 (FAR = 1.52)
ResNet-18	Cosine	61.59 (FAR = 1.45)	81.42 (FAR = 5.40)	59.25 (FAR = 0.56)	79.66 (FAR = 3.99)
ResNet-50	Cosine	50.90 (FAR = 1.19)	76.84 (FAR = 4.90)	34.99	70.32 (FAR = 2.57)
**Sejong Face Dataset**					
**CNN Model**	**Distance Function**	**Without Glasses**		**With Glasses**	
		**TAR@** **FAR 0.1% ^3^**	**TAR@** **FAR 1% ^3^**	**TAR@** **FAR 0.1% ^3^**	**TAR@** **FAR 1% ^3^**
MobileNetv2	Cosine	35.41	62.10	7.11	24.48
ResNet-18	Cosine	48.46	78.30 (FAR = 1.90)	44.53 (FAR = 0.54)	80.08 (FAR = 8.79)
ShuffleNet	Cosine	18.36	58.62	50.57 (FAR = 4.29)	80.75 (FAR = 20.25)

^1^ This dataset contains the IOE_WAT and D4FLY datasets. ^2^ This is only the IOE_WAT dataset that contains subjects wearing glasses. ^3^ The values located in parentheses indicate the FAR for the test dataset only if it differs significantly from the FAR calculated for the training dataset.

**Table 5:** Performance of Triplet algorithms for the joint ^1^, Speaking Faces, and Sejong Face datasets for a thermal anchor image.

Joint Dataset ^1^					
CNN Model	Distance Function	Without Glasses		With Glasses ^2^	
		TAR@ FAR 0.1% ^3^	TAR@ FAR 1% ^3^	TAR@ FAR 0.1% ^3^	TAR@ FAR 1% ^3^
Inceptionv3	Cosine	17.36 (FAR = 1.66)	31.94 (FAR = 6.17)	3.54 (FAR = 1.33)	28.54 (FAR = 12.50)
ResNet-50	Cosine	11.03 (FAR = 0.85)	25.42 (FAR = 3.39)	1.14 (FAR = 0.38)	23.61 (FAR = 11.36)
VGG19	Correlation	10.03	25.96	8.27 (FAR = 1.01)	22.16 (FAR = 5.43)
**Speaking Faces Dataset**					
**CNN Model**	**Distance Function**	**Without Glasses**		**With Glasses**	
		**TAR@** **FAR 0.1% ^3^**	**TAR@** **FAR 1% ^3^**	**TAR@** **FAR 0.1% ^3^**	**TAR@** **FAR 1% ^3^**
Inceptionv3	Cosine	18.75	38.71 (FAR = 10.29)	23.11 (FAR = 13.98)	44.46 (FAR = 32.32)
ResNet-18	Cosine	10.45	26.69 (FAR = 7.70)	10.79 (FAR = 4.18)	28.23 (FAR = 16.30)
ResNet-101	Cosine	21.26	33.67 (FAR = 8.94)	8.24 (FAR = 3.64)	18.86 (FAR = 9.98)
**Sejong Face Dataset**					
**CNN Model**	**Distance Function**	**Without Glasses**		**With Glasses**	
		**TAR@** **FAR 0.1% ^3^**	**TAR@** **FAR 1% ^3^**	**TAR@** **FAR 0.1% ^3^**	**TAR@** **FAR 1% ^3^**
Inceptionv3	Cosine	9.25 (FAR = 2.10)	21.51 (FAR = 8.33)	40.71 (FAR = 29.11)	67.54 (FAR = 54.46)
MobileNetv2	Spearman	3.08	10.82 (FAR = 2.30)	0.13	1.48
VGG16	Correlation	5.77 (FAR = 0.59)	11.08 (FAR = 1.90)	0.67	5.70

^1^ This dataset contains the IOE_WAT and D4FLY datasets. ^2^ This is only the IOE_WAT dataset that contains subjects wearing glasses. ^3^ The values located in parentheses indicate the FAR for the test dataset only if it differs significantly from the FAR calculated for the training dataset.

**Table 6:** Performance of VTI for the joint, Speaking Faces, and Sejong Face datasets.

Joint Dataset ^1^				
	Without Glasses		With Glasses ^2^	
CNN Model	TAR@ FAR 0.1%	TAR@ FAR1%	TAR@ FAR 0.1%	TAR@ FAR1%
DenseNet-201	1.29	2.76	0.22	2.75
Inceptionv3	0.12	0.87	0.19	2.08
VGG19	0.01	0.98	0.63	2.18
**Speaking Faces Dataset**				
	**Without Glasses**		**With Glasses**	
**CNN Model**	**TAR@** **FAR 0.1%**	**TAR@** **FAR1%**	**TAR@** **FAR 0.1%**	**TAR@** **FAR1%**
DenseNet-201	0.04	0.80	0.39	2.85
Inceptionv3	0.26	1.61	0.63	3.04
ShuffleNet	0.25	1.51	0.91	3.96
**Sejong Face Dataset**				
	**Without Glasses**		**With Glasses**	
**CNN Model**	**TAR@** **FAR 0.1%**	**TAR@** **FAR1%**	**TAR@** **FAR 0.1%**	**TAR@** **FAR1%**
Inceptionv3	1.61	4.66	0.07	0.80
ResNet-101	0.75	3.61	0.17	1.37
ShuffleNet	0.03	1.61	0.34	3.42

^1^ This dataset contains the IOE_WAT and D4FLY datasets. ^2^ This is only the IOE_WAT dataset that contains subjects wearing glasses.

**Table 7:** Performance of triple triplet algorithms for the IOE_WAT, Speaking Faces, and Sejong Face datasets for a visible anchor image.

Joint Dataset ^1^					
CNN Model	Distance Function	Without Glasses		With Glasses ^2^	
		TAR@ FAR 0.1% ^3^	TAR@ FAR 1% ^3^	TAR@ FAR 0.1% ^3^	TAR@ FAR 1% ^3^
ResNet-18 ResNet-50 ShuffleNet	Cosine	60.76	76.93	43.75	67.17
**Speaking Faces Dataset**					
**CNN Model**	**Distance Function**	**Without Glasses**		**With Glasses ^2^**	
		**TAR@** **FAR 0.1% ^3^**	**TAR@** **FAR 1% ^3^**	**TAR@** **FAR 0.1% ^3^**	**TAR@** **FAR 1% ^3^**
ResNet-18 ResNet-50 ShuffleNet	Cosine	77.47 (FAR = 1.69)	90.61 (FAR = 6.74)	70.79	87.52 (FAR = 2.75)
**Sejong Face Dataset**					
**CNN Model**	**Distance Function**	**Without Glasses**		**With Glasses ^2^**	
		**TAR@** **FAR 0.1% ^3^**	**TAR@** **FAR 1% ^3^**	**TAR@** **FAR 0.1% ^3^**	**TAR@** **FAR 1% ^3^**
ResNet-18 ResNet-50 ShuffleNet	Cosine	50.89	78.23	72.84 (FAR = 4.36)	91.62 (FAR = 19.11)

^1^ This dataset contains the IOE_WAT and D4FLY datasets. ^2^ This is only the IOE_WAT dataset that contains subjects wearing glasses. ^3^ The values located in parentheses indicate the FAR for the test dataset only if it differs significantly from the FAR calculated for the training dataset.

**Table 8:** Performance of triple triplet algorithms according to different head positions.

Triplet (Visible Anchor Image) ^2^			
Dataset	Position of Head	TAR@FAR0.1% ^1^	TAR@FAR1% ^1^
D4FLY	frontal	59.10	73.64
	not frontal	33.93	53.57
IOEpart1	frontal	62.50	89.45
	not frontal	51.56	76.56
IOEpart2	frontal	54.38	65.94
	not frontal	23.50	44.25
Speaking Faces	frontal	69.14	85.94
	not frontal	55.55	77.81
IOE_WAT (glasses)	frontal	36.36	63.07
	not frontal	23.52	45.80
Speaking Faces (glasses)	frontal	68.85	85.94
	not frontal	51.58	74.65
**Triple Triplet ^3^**			
**Dataset**	**Position of Head**	**TAR@FAR0.1% ^1^**	**TAR@FAR1% ^1^**
D4FLY	frontal	68.34	80.57
	not frontal	49.29	66.96
IOEpart1	frontal	89.06	93.36
	not frontal	69.38	83.44
IOEpart2	frontal	64.38	82.50
	not frontal	35.00	64.00
Speaking Faces	frontal	84.51	92.76
	not frontal	71.84	88.89
IOE_WAT (glasses)	frontal	53.69	73.58
	not frontal	35.80	62.05
Speaking Faces (glasses)	frontal	81.25	91.24
	not frontal	62.42	84.54

^1^ The values located in parentheses indicate the FAR for the test dataset only if it differs significantly from the FAR calculated for the training dataset. ^2^ ResNet-18 model and cosine distance. ^3^ ResNet-18, ResNet-50 and ShuffleNet with cosine distance.

**Table 9:** Gender-divided performance of the triplet-based algorithm with a visible anchor image.

Joint Dataset (ResNet-50 and Cosine Distance)		
Gender	TAR@ FAR 0.1%	TAR@ FAR 1%
Female	38.77	60.76
Male	46.18	71.47
**Speaking Face (MobileNetv2 and Cosine Distance)**		
Female	52.99	73.13
Male	38.24	61.54
**Sejong Face (MobileNetv2 and Cosine Distance)**		
Female	30.20	52.80
Male	37.95	66.63

**Table 10:** Gender-divided performance of the triple triplet method with a thermal anchor image.

Joint Dataset		
Gender	TAR@ FAR 0.1%	TAR@ FAR 1%
**Female**	66.32	77.31
**Male**	57.99	76.74
**Speaking Face**		
**Female**	83.00	93.37
**Male**	72.38	88.07
**Sejong Face**		
**Female**	32.00	64.00
**Male**	60.10	85.17

**Table 11:** Average processing times.

Verification Method	Time (s)
Triplet (with ResNet-18)	1.06
Triple triplet	2.19

**Table 12:** Results of the ablation study for the triple and triple triplet methods with a visible anchor image.

Joint Dataset ^1^					
Triplet					
CNN Model	Distance Function	Thermal Training Dataset		Visible Training Dataset	
		TAR@ FAR 0.1%	TAR@ FAR 1%	TAR@ FAR 0.1%	TAR@ FAR 1%
ResNet-18	Cosine	16.13	38.19	14.20	31.13
**Triple Triplet**					
**CNN Model**	**Distance Function**	**Thermal Training Dataset**		**Visible Training Dataset**	
		**TAR@** **FAR 0.1%**	**TAR@** **FAR 1%**	**TAR@** **FAR 0.1%**	**TAR@** **FAR 1%**
ResNet-18 ResNet-50 ShuffleNet	Cosine	26.58	52.08	22.65	41.20
**Speaking Faces Dataset**					
**Triplet**					
**CNN Model**	**Distance Function**	**Thermal Training Dataset**		**Visible Training Dataset**	
		**TAR@** **FAR 0.1% ^2^**	**TAR@** **FAR 1% ^2^**	**TAR@** **FAR 0.1%**	**TAR@** **FAR 1% ^2^**
ResNet-18	Cosine	49.06 (FAR = 2.16)	77.55 (FAR = 10.86)	39.79	68.03 (FAR = 1.48)
**Triple Triplet**					
**CNN Model**	**Distance Function**	**Thermal Training Dataset**		**Visible Training Dataset**	
		**TAR@** **FAR 0.1% ^2^**	**TAR@** **FAR 1% ^2^**	**TAR@** **FAR 0.1%**	**TAR@** **FAR 1%**
ResNet-18 ResNet-50 ShuffleNet	Cosine	53.14 (FAR = 0.86)	85.50 (FAR = 11.22)	45.71	73.76
**Sejong Face Dataset**					
**Triplet**					
**CNN Model**	**Distance Function**	**Thermal Training Dataset**		**Visible Training Dataset**	
		**TAR@** **FAR 0.1%**	**TAR@** **FAR 1%**	**TAR@** **FAR 0.1%**	**TAR@** **FAR 1%**
ResNet-18	Cosine	15.80	51.74	22.75	52.00
**Triple Triplet**					
**CNN Model**	**Distance Function**	**Thermal Training Dataset**		**Visible Training Dataset**	
		**TAR@** **FAR 0.1%**	**TAR@** **FAR 1% ^2^**	**TAR@** **FAR 0.1%**	**TAR@** **FAR 1%**
ResNet-18 ResNet-50 ShuffleNet	Cosine	19.48	68.66 (FAR = 3.15)	12.26	42.56

^1^ This dataset contains the IOE_WAT and D4FLY datasets. ^2^ The values located in parentheses indicate the FAR for the test dataset only if it differs significantly from the FAR calculated for the training dataset.

## Data Availability

Not applicable.
